# Développement d’un score prédictif de malnutrition aiguë sévère chez les enfants de moins de 5 ans

**DOI:** 10.11604/pamj.2018.29.185.13713

**Published:** 2018-04-02

**Authors:** Olivier Mukuku, Augustin Mulangu Mutombo, Lewis Kipili Kamona, Toni Kasole Lubala, Paul Makan Mawaw, Michel Ntani Aloni, Stanislas Okitotsho Wembonyama, Oscar Numbi Luboya

**Affiliations:** 1 Institut Supérieur des Techniques Médicales de Lubumbashi, République Démocratique du Congo; 2 Département de Pédiatrie, Faculté de Médecine, Université de Lubumbashi, République Démocratique du Congo; 3 Département de Santé Publique, Faculté de Médecine, Université de Lubumbashi, République Démocratique du Congo

**Keywords:** Malnutrition aiguë sévère, score prédictif, facteurs de risque, dépistage, enfant

## Abstract

**Introduction:**

L’objectif de cette étude est de développer un score prédictif de la malnutrition aiguë sévère (MAS) chez les enfants de 6 à 59 mois.

**Méthodes:**

Etude cas-témoins: les cas (n = 263) étaient les enfants de 6 à 59 mois admis à l’hôpital Sendwe de Lubumbashi pour une MAS; les témoins (n = 263) étaient les enfants de même âge admis dans le même hôpital pour une pathologie autre que la MAS. Nous avons procédé par une analyse univariée puis multivariée. La discrimination du score était évaluée à l’aide de la courbe ROC et du C-index.

**Résultats:**

Après modélisation logistique, neuf critères ressortent comme facteurs prédictifs de MAS: le faible poids à la naissance, la diarrhée à répétition/chronique, le nombre de repas journaliers < 3, l’âge d’arrêt d’allaitement maternel < 6 mois, l’âge d’introduction d’alimentation de complément < 6 mois, l’âge maternel < 25 ans, la parité < 5, l’antécédent familial de malnutrition et le nombre d’enfants âgés de moins de 5 ans dans la fratrie ≥ 3. L’aire sous la courbe ROC est de 0,9685, la sensibilité de 93,54%, la spécificité de 93,16%, la valeur prédictive positive de 93,18% et le rapport de vraisemblance positif de 6,84%.

**Conclusion:**

Nous proposons un score prédictif du risque de survenue de MAS dans une population de moins de 5 ans. Ce score prédictif de MAS serait un outil clinique utile et simple pour cibler la population à risque, limiter les taux élevés de malnutrition et réduire la morbidité et la mortalité infanto-juvénile enregistrés dans les pays en développement.

## Introduction

Dans les pays en développement, les pratiques d’alimentation sont très souvent inadéquates et incompatibles avec les recommandations de l’Organisation mondiale de la santé (OMS) [1]. Un mauvais état nutritionnel pendant la petite enfance a également des répercussions sur la santé à l’âge adulte [2]. L’OMS estimait que la malnutrition aiguë sévère (MAS) affecte environ 16 millions d’enfants de moins de 5 ans [3]. Bien que connue pour être un problème majeur de santé publique dans les pays à faibles revenus, la malnutrition contribue de manière significative à la mortalité chez les enfants de moins de cinq ans et en 2011, il a été estimé qu’environ 45% des décès d´enfants serait attribuée à la malnutrition [4,5]. La République Démocratique du Congo fait partie des pays comptant un taux élevé de mortalité chez les enfants de moins de 5 ans [6] et la malnutrition est l’une des principales causes de décès dans ces pays en s’associant à d’autres maladies comme la diarrhée, la pneumonie et le paludisme; maladies plus fréquentes chez l’enfant âgé de moins de 5 ans [5]. La malnutrition infantile est influencée par des facteurs multidimensionnels. Selon Kikafunda, les facteurs qui influent sur la malnutrition infantile dans les pays en développement sont divisés en trois groupes: les facteurs maternels, les facteurs alimentaires et socio-environnementaux et les facteurs économiques [7]. Un certain nombre d´études ont démontré que la malnutrition infantile est fortement ancrée dans la pauvreté [8-11]. Cependant, la relation entre la pauvreté et la malnutrition infantile est assez complexe. La malnutrition affecte aussi les ménages pauvres que les ménages non pauvres [12,13]. Les revenus élevés des ménages ne peuvent pas garantir un résultat nutritionnel satisfaisant des enfants si les ménages manquent les soins hygiéniques, la qualité alimentaire et l´accès aux soins de santé [14-16]. La présente étude vise à développer un score prédictif de MAS chez les enfants de 6 à 59 mois.

## Méthodes

Il s’agit d’une étude cas-témoins conduite à l’hôpital Jason Sendwe à Lubumbashi (RDC) ayant porté sur les enfants âgés de 6 à 59 mois entre le 1^er^ Janvier 2011 au 31 Décembre 2012. Ces enfants sont issus d’une population qui vit dans une zone non conflictuelle (pas de conflits armés). Les cas étaient constitués des enfants âgés de 6 à 59 mois admis à l’hôpital pour une MAS qui était définie par un z-score poids/taille < -3 (calculé à l’aide du logiciel WHO Anthro 2011 version 3.2.2) ou un périmètre brachial < 115 mm ou la présence d’œdèmes bilatéraux de malnutrition [17,18]. La prise en charge de la MAS suit les étapes du guide de l’OMS [19] adopté par le programme de nutrition de la RDC. Les témoins étaient composés des enfants de même âge consultés dans le même hôpital pour une pathologie autre que la MAS. Les cas et les témoins, tous âgés de 6 à 59 mois, ont été inclus de façon exhaustive et prospective après un consentement oral libre et éclairé de leurs parents et l’appariement était de 1:1 en fonction de la date de consultation. Le nombre de sujets inclus dans l’étude étaient de 263 cas et 263 témoins. Nous avons exclu de l’étude les sujets dont le statut VIH était positif ou inconnu, ainsi que ceux qui avaient une pathologie pouvant influencer la croissance ou l’évolution en cours d’hospitalisation. Il s’agissait entre autres des déformations rachidiennes ou des membres inférieurs, des cardiopathies, des néphropathies, des neuropathies chroniques, des anomalies digestives avec un syndrome de malabsorption ainsi que la drépanocytose. L’étude a bénéficié d’une autorisation du comité d’éthique et de la recherche de la faculté de médecine de l’Université de Lubumbashi.

### Variables d’étude

Nous avons étudiés les variables suivantes: 1) *Les caractéristiques et antécédents personnels de l’enfant:* l’âge, le sexe, la notion de faible poids à la naissance (défini par un poids de naissance < 2500 grammes), la diarrhée à répétition ou chronique, le suivi de consultations préscolaires (CPS); 2) *Les pratiques alimentaires:* l’âge d’introduction d’aliments de complément (considérée précoce lorsque cette introduction était faite avant l’âge de 6 mois), l’âge d’arrêt d’allaitement maternel (considérée précoce lorsque cet arrêt était fait avant l’âge de 6 mois), le nombre de repas journaliers [20]; 3) *Les antécédents maternels et paternels:* nous avons étudié, *chez la mère*, l’âge, la parité, le statut matrimonial (vivant seule (singleton) ou en union), la profession (répartie en occupée et sans occupation), le niveau de scolarité (était considérée de bas niveau lorsque la mère avait atteint au plus 6 années d’études) et *chez le père,* la profession (répartie en occupé et sans occupation), le niveau de scolarité (était considéré de bas niveau lorsque le père avait atteint au plus 6 ans d’études). En plus, nous avons recherché l’existence de parents c’est-à-dire préciser si l’un ou l’autre ou les deux parents biologiques étaient en vie et les enfants étaient répartis en orphelins et non orphelins; 4) *Les antécédents familiaux:* l’antécédent familial de malnutrition, le nombre d’enfants de moins de 5 ans dans la famille, la taille de la famille (définie par le nombre de personnes constituant la famille et habitant sous le même toit).

### Analyses statistiques

Le logiciel STATA 12 a été utilisé pour les différentes analyses statistiques. Pour déterminer les facteurs prédictifs de MAS, nous avons effectué une analyse unifactorielle en utilisant le test du Chi^2^ ou le test exact de Fisher, puis nous avons réalisé une analyse multifactorielle avec une régression logistique. Les variables ayant un degré de signification inférieur à 0,05 dans l’analyse unifactorielle ont été inclues dans le modèle multifactoriel en utilisant la méthode pas à pas. Nous avons retenu dans le modèle final les variables dont le seuil de signification était inférieur à 0,05. La discrimination du score était évaluée à l’aide de la courbe ROC et du C-index et la calibration du score selon le test d’Hosmer-Lemeshow. Nous avons déterminé la sensibilité, la spécificité et le pourcentage de cas correctement classés par rapport au C-index. L’évaluation de la robustesse des coefficients du modèle était faite par bootstrap. Le score prédictif du risque était déduit au terme de l’analyse statistique et était établi en assignant des points à chaque facteur de risque retenu dans le modèle logistique. Pour le rendre simple à utiliser, le score était réalisé par l’utilisation de valeurs arrondies de ces coefficients. Les probabilités de risque de MAS en fonction des valeurs du score construit ont été calculées.

### Considérations éthiques

La participation à l’étude était confidentielle après le consentement libre et éclairé des mères. Les données ont été analysées dans la plus stricte confidentialité. Après chaque entretien, des informations/conseils sur les pratiques alimentaires ont été donnés. Le comité d’éthique médical de l´Université de Lubumbashi a approuvé ce projet.

## Résultats

Le Tableau 1 montre qu´il y avait une association statistiquement significative entre la MAS et le poids de naissance, la notion de diarrhée à répétition/chronique, l’âge d’arrêt d’allaitement maternel, l’âge d’introduction d’aliments de complément, le nombre de repas quotidiens, les consultations préscolaires, l’existence de parents, l’antécédent de malnutrition dans la famille, le nombre d’enfants de moins de 5 ans dans la fratrie, la taille de la famille, l’âge maternel, la parité, le statut matrimonial de la mère, la profession maternelle, le niveau de scolarité maternel, la profession paternelle et le niveau de scolarité paternel. La MAS était plus importante quand l´enfant était né avec un faible poids (< 2500 grammes), en présence de la notion de diarrhée à répétition ou chronique, en présence d’un âge d’arrêt d’allaitement maternel inférieur à 6 mois, en présence d’un âge d’introduction d’aliments de complément inférieur 6 mois, quand le nombre de repas journaliers était < 3, quand les CPS n’étaient pas suivies, quand l’enfant était orphelin d’un ou de deux parents, en présence d’antécédent de malnutrition familiale, quand le nombre d’enfants de moins de 5 ans était ≥ 3, quand la taille de famille était > 6, en présence d’un âge maternel < 25 ans, en présence d’une parité < 5, quand la mère vivait seule, en présence des parents de bas niveau de scolarité et sans emploi.

**Tableau 1:** Analyse univariée de principaux facteurs de risque de survenue de la malnutrition chez les enfants de 6 à 59 mois à Lubumbashi (RDC)

Variables	MAS présente(n = 263)	MAS absente(n = 263)	OR brut [IC95%]	p
	n (%)	n (%)		
Age <24 mois	187 (71,1)	182 (69,2)	1,09 [0,75-1,59]	0,6340
Sexe masculin	161 (61,2)	164 (62,4)	1,04 [0,73-1,49]	0,7879
Faible poids de naissance	124 (47,2)	20 (7,6)	10,83 [6,46-18,16]	< 0,000001
Diarrhée à répétition/chronique	189 (71,9)	28 (10,7)	21,43 [13,32-34,47]	< 0,000001
Age d’arrêt d’allaitement maternel <6 mois	24 (9,1)	3 (1,2)	8,70 [2,58-29,27]	< 0,0001
Age d’introduction d’aliments de complément <6 mois	234 (89,0)	121 (46,0)	9,46 [6,00-14,93]	< 0,000001
<3 repas journaliers	230 (87,5)	58 (22,1)	24,63 [15,44-39,29]	< 0,000001
Non suivi de consultations préscolaires	193 (73,4)	22 (8,4)	30,20 [18,04-50,55]	< 0,000001
Orphelin d’un ou de deux parents	46 (17,5)	10 (3,8)	5,36 [2,64-10,88]	< 0,00001
Présence d’antécédent de malnutrition dans la famille	136 (51,7)	10 (3,8)	27,09 [13,77-53,29]	< 0,000001
≥3 enfants de moins de 5 ans dans la fratrie	94 (35,7)	7 (2,6)	20,34 [9,21-44,91]	< 0,000001
>6 personnes dans la famille	119 (45,2)	40 (15,2)	4,60 [3,04-6,97]	< 0,000001
Age maternel <25 ans	81 (30,8)	9 (3,4)	12,56 [6,14-25,66]	< 0,000001
Parité <5	117 (44,5)	42 (16,0)	4,21 [2,79-6,35]	< 0,000001
Mère singleton	98 (37,3)	18 (6,8)	8,08 [4,71-13,87]	< 0,000001
Mère sans emploi	242 (92,0)	124 (47,2)	12,91 [7,77-21,45]	< 0,000001
Mère de bas niveau de scolarité	174 (66,2)	39 (14,8)	11,22 [7,33-17,18]	< 0,000001
Père sans emploi	182 (69,2)	140 (53,2)	1,97 [1,38-2,82]	0,0002
Père de bas niveau de scolarité	108 (41,1)	10 (3,8)	17,62 [8,94-34,72]	< 0,000001

**Figure 1:**
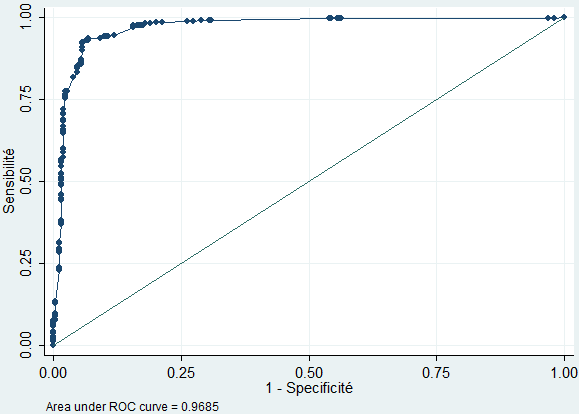
Courbe ROC montrant les performances du score de MUKUKU prédictif de MAS

Après modélisation logistique, neuf critères ressortent comme facteurs prédictifs de MAS: le faible poids à la naissance, la diarrhée à répétition ou chronique, un nombre de repas journaliers inférieur à 3, âge d’arrêt d’allaitement maternel inférieur à 6 mois, un âge d’introduction d’aliments de complément inférieur à 6 mois, un âge maternel inférieur à 25 ans, une parité inférieur à 5, un antécédent familial de malnutrition, un nombre d’enfants âgés de moins de 5 ans dans la fratrie = 3 (Tableau 2). Chaque facteur de risque a été pondéré par un coefficient de régression représentant le poids de la variable dans le calcul du score. L’ensemble des scores obtenus est illustré dans le Tableau 3. Le score prédictif de la MAS a été construit à partir du modèle logistique (Tableau 3). L’aire sous la courbe ROC est de 0,9685 (Figure 1), laquelle courbe montre une discrimination exceptionnelle en ce qui concerne sa capacité de discriminer les enfants qui vont présenter la MAS de ceux qui ne vont pas la présenter.

**Tableau 2:** Modèle de régression logistique du risque de MAS et score de facteurs prédictifs

Variable	OR ajusté	ICà95%	Coefficient	Score
Faible poids de naissance	2,72	1,18-6,26	1,00	1
Diarrhée à répétition/chronique	10,34	4,94-21,62	2,33	2
Nombre de repas journaliers <3	9,86	4,66-20,85	2,28	2
Age d’arrêt d’allaitement maternel <6 mois	9,08	1,63-50,62	2,20	2
Age d’introduction d’aliments de complément <6 mois	3,19	1,38-7,35	1,16	1
Age maternel <25 ans	16,60	5,92-46,56	2,80	3
Parité <5	6,03	2,27-16,04	1,79	2
Antécédent familial de malnutrition	24,89	8,77-70,63	3,21	3
Nombre d’enfants de moins de 5 ans ≥3	5,39	1,66-17,47	1,68	2

**Tableau 3:** Probabilité de la MAS en fonction du score selon le modèle de régression logistique

Score obtenu	Probabilité de la MAS^+^
0	0,22%
1	0,58%
2	1,53%
3	3,93%
4	9,74%
5	22,14%
6	42,82%
7	66,36%
8	83,86%
9	93,19%
10	97,30%
11	98,95%
12	99,60%
13	99,84%
14	99,94%
15	99,97%
16	99,99%
17	99,99%
18	99,99%

+ obtenu à partir de la formule: p=1/1 + exp (6,1 + 0,9685 x score)

**Figure 2:**
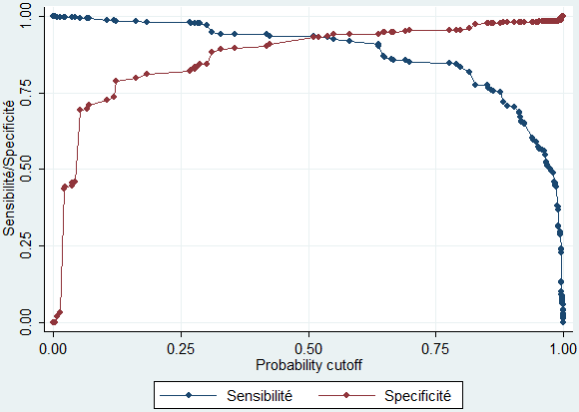
Sensibilité et spécificité du score de MUKUKU prédictif de MAS

La présence de ces neuf critères affecte un certain nombre de points dont le total est de 18 points. Pour chaque enfant, le score varie de 0 à 18 et au plus il est élevé, au plus le risque de MAS est élevé. Les probabilités de risque de MAS en fonction des valeurs du score construit ont été calculées et sont présentées dans le tableau 3. Un score < 6 définit les enfants à faible risque de MAS, un score entre 6 et 8 points définit un risque modéré de MAS et un score > 8 points présente un risque élevé de MAS. Ainsi une sensibilité de 93,54% a été obtenue pour une spécificité de 93,16%, ce qui signifie qu’avec ce seuil, seuls 6,46% des enfants présentant la MAS n’obtenaient pas un score positif et 6,84% des enfants ne présentant pas la MAS obtenaient un score faussement positif (**Figure 2**). La valeur prédictive positive était de 93,18%.

## Discussion

Dans la présente étude, nous avons retrouvé en analyse multifactorielle les facteurs de risque de MAS chez les enfants de moins de 5 ans, lesquels facteurs qui sont globalement concordants avec ceux rapportés dans plusieurs études menées dans les pays en développement. En conformité avec d’autres études [21-26], le faible poids de naissance a été trouvé comme étant un facteur de MAS. Ceci pourrait s’expliquer par le fait que le faible poids de naissance dans notre milieu résulte le plus souvent d’une malnutrition maternelle [27], ce qui laisse sous-entendre que les conditions dans lesquelles l’enfant vivra désormais sont précaires du point de vue de la sécurité alimentaire mais aussi des pratiques d’alimentation et d’hygiène du milieu. Il n’est donc pas étonnant qu’un enfant déjà malnutri avant la naissance et vivant dans ces conditions voit sa malnutrition persister ou s’aggraver. Dans notre série, l’antécédent de diarrhée à répétition ou chronique a été associée significativement à la survenue de la malnutrition; résultat similaire à ceux des plusieurs auteurs [28-30]. Ceci peut s’expliquer par le fait que la diarrhée s’accompagne d’une baisse de l’appétit et d’une diminution de l’absorption des nutriments au niveau du tube digestif, réalisant ainsi un véritable cercle vicieux diarrhée-malnutrition et plus largement infection-malnutrition. Un mauvais état nutritionnel augmente en effet la gravité, la durée et l’incidence des épisodes diarrhéiques.

Comme constaté dans les études antérieures [30-35], notre étude a également montré que l’arrêt précoce d’allaitement maternel et l’introduction précoce de l’alimentation de complément étaient associés significativement à la MAS. Cet arrêt précoce est souvent décidé brusquement au cours d’une maladie de l’enfant ou en raison d’une nouvelle grossesse perturbant ainsi l’équilibre nutritionnel de l’enfant et entrainant par conséquent un état de malnutrition. Une étude menée en Chine avait montré que l’introduction d’autres aliments avant l’âge de 6 mois augmentait la prévalence des pathologies diarrhéiques et de pneumonie [36]. Nous avons noté une association significative entre moins de trois repas par jour et la MAS. Une étude menée au Bénin avait mis en évidence l’association significative entre la malnutrition et le défaut quantitatif de la ration alimentaire de 24 dernières heures [37]. Ceci peut s’expliquer par le fait qu’une bonne alimentation doit respecter quelques conditions dont une bonne qualité, une quantité suffisante et une fréquence de prise des repas acceptable.

En outre, le nombre élevé d’enfants de moins de 5 ans dans la fratrie a été trouvé comme facteur prédictif de malnutrition infantile même après analyses multivariées. Une conclusion similaire a été rapportée dans plusieurs études [24,38,39]. Cette association peut s’expliquer par le fait d’avoir qu’un grand nombre d’enfants en bas âge exige beaucoup d’attention et de ressources pour l’alimentation et les soins de santé. L’augmentation du nombre d’enfants dans la famille est une charge lourde sur les ressources des ménages en particulier sur la nourriture et les finances réduisant ainsi le temps et la qualité des soins reçus par les enfants [24,40]. Nous avons mis en évidence une association entre l’antécédent familial de malnutrition et influence la survenue de la MAS, ce qui est compatible avec les résultats d’études menées ailleurs [37,41]. Ceci peut s’expliquer par le fait qu’un antécédent familial de malnutrition peut traduire les mauvaises conditions de vie et d’alimentation dans cette famille, exposant ainsi tous les autres membres de famille au même risque et plus particulièrement les jeunes enfants.

Nous avons trouvé que le jeune âge maternel (< 25 ans) et la parité faible (< 5) influaient sur la survenue de la MAS. Ayaya trouvait aussi que l’âge maternel inférieur à 25 ans est un facteur de risque de la malnutrition sévère [42]. D’autres auteurs soulignent également que l’âge maternel et la parité sont des facteurs positifs et significatifs de l’état nutritionnel des enfants et rapportent que les enfants nés des jeunes mères sont plus susceptibles de souffrir des problèmes de santé que les enfants nés de femmes adultes [43]. Cette association peut s’expliquer par les difficultés qu’éprouvent généralement les nouvelles mères (surtout jeunes) à assumer à la fois un ménage, un enfant, la santé de l’enfant et à prodiguer des soins adéquats à ses enfants (souvent le premier) surtout lors du sevrage. Le sevrage est souvent mal conduit, d’où la détérioration de l’état nutritionnel des enfants pendant cette période. Ces mères présentent un faible niveau de connaissance des besoins alimentaires de l’enfant des valeurs nutritives des différents types d’aliments donnés à l’enfant. Ces informations sont souvent données aux mères lors des consultations de suivi de croissance des enfants.

Nous avons identifié différentes variables qui nous ont permis d’établir le score prédictif de risque de MAS chez les enfants de moins de 5 ans. L’analyse de la courbe ROC nous a conduits à définir un seuil à la fois suffisamment sensible et spécifique pour dépister les enfants à risque de présenter une MAS. Ce seuil reste limité à une sensibilité de 93,54% pour une spécificité de 93,16%. Notre score permet de prédire la MAS chez plus de 9 sur 10 enfants malnutris, mais classe faussement comme malnutris 6,84% des enfants bien nourris. Les résultats de notre étude proviennent de l’analyse des données récoltées sur la population d’un seul hôpital. Néanmoins, ce dernier reçoit les enfants malades référés de presque toute la la partie Sud-Est de la RDC. Cette étude devra également être menée dans d’autres régions de la RDC comme en Afrique à court terme pour évaluer et valider les performances de ce modèle sur des populations différentes (transportabilité). De ce fait, le modèle présenté ici n’a pas la prétention d’avoir une validité universelle. Cet outil proposé trouve son importance dans son utilisation dans le dépistage de risque de MAS avant toute survenue de la MAS. Les enfants pourront être soumis à cet outil lors de campagnes de vaccination dans la communauté ou lors de consultations préscolaires. Ceux qui présenteront un risque élevé pourront être suivis et leurs mères bénéficieront des conseils et des informations sur les besoins alimentaires de l’enfant ainsi que les valeurs nutritives des différents types d’aliments donnés à l’enfant.

## Conclusion

Cette étude multifactorielle des variables favorisant la MAS permet de proposer un score prédictif de la survenue qui repose sur des covariables faciles à recueillir avant toute hospitalisation voire même au cours des consultations préscolaires de routine. Aucun score publié n’est adapté pour prédire le risque de survenue de MAS dans une population de moins de 5 ans dans les pays en développement. Nous proposons donc un score simple et performant, prédictif du risque de MAS qui nécessitera une étude de validation externe c’est-à-dire dans une population différente de celle qui a servi à l’établir. Ce score prédictif de MAS serait un outil clinique utile et simple pour cibler la population à risque, limiter les taux élevés de malnutrition et réduire la morbidité et la mortalité infanto-juvénile enregistrés dans les pays en développement.

### Etat des connaissances actuelles sur le sujet

- La République Démocratique du Congo fait partie des pays comptant un taux élevé de mortalité chez les enfants de moins de 5 ans et la malnutrition est l’une des principales causes de décès dans notre pays;
- Les facteurs qui influent sur la malnutrition infantile dans les pays en développement sont divisés en trois groupes: les facteurs maternels, les facteurs alimentaires et socio-environnementaux et les facteurs économiques.

### Contribution de notre étude à la connaissance

- L’étude proposée est la première étude dans notre ville voire dans notre pays, intégrant une analyse multivariée permettant d’identifier les facteurs de risque de la malnutrition aiguë sévère chez les enfants de moins de 5 ans dans notre contexte, à Lubumbashi, République Démocratique du Congo;
- Elle est également la première à proposer un outil trouvant son importance dans son utilisation dans le dépistage de risque de malnutrition aiguë sévère avant toute survenue de cette dernière dans notre contexte.
